# Nutrition disorders and related conditions—Prevalence, overlap and relation to one year survival in geriatric patients

**DOI:** 10.1016/j.jnha.2025.100682

**Published:** 2025-09-20

**Authors:** Frida Ostonen Peelen, Maria Enge, Rikke Lundsgaard Nielsen, Anne Marie Beck, Ann Ödlund Olin, Tommy Cederholm, Anne-Marie Boström, Ingvild Paur

**Affiliations:** a Theme Inflammation and Aging, Nursing Unit Aging, Karolinska University Hospital, Huddinge, Hälsovägen 13, 141 86 Stockholm, Sweden; b Department of Geriatric Medicine, Jakobsbergsgeriatriken, Jakobsbergs Sjukhus, Birgittavägen 2a, 177 31 Järfälla, Sweden; c Järfälla Rehabilitation, Stockholm County Healthcare Services, Jakobsbergs Sjukhus, Birgittavägen 2a, 177 31 Järfälla, Sweden; d Department of Clinical Research, Copenhagen University Hospital Amager and Hvidovre, Kettegård Allé 18, 2650 Hvidovre, Denmark; e Dietetic and Nutritional Research Unit EATEN, Herlev and Gentofte University Hospital, Borgmester Ib Juuls Vej 1, 2730 Herlev, Denmark; f Department of Clinical Science, Intervention and Technology, Karolinska Institute, 171 77 Stockholm, Sweden; g Theme Inflammation and Aging, Medical Unit Aging, Karolinska University Hospital, Huddinge, 141 86 Stockholm, Sweden; h Clinical Nutrition and Metabolism, Department of Public Health and Caring Sciences, Uppsala University, 751 22 Uppsala, Sweden; i Karolinska Institute, Department of Neurobiology, Care Sciences and Society, Division of Nursing, 141 83 Huddinge, Sweden; j Stockholms Sjukhem, Research and Development Unit, Box 12230, 102 25 Stockholm, Sweden; k Norwegian National Network for Disease-related Malnutrition, Domus Medica, Sognsvannsveien 9, 0372 Oslo, Norway; l Department of Clinical Service, Division of Cancer Medicine, Oslo University Hospital, Box 4950 Nydalen, 0424 Oslo, Norway; m Institute for Clinical Medicine, Clinical Nutrition Research Group, UiT the Arctic University of Norway, Box 6050 Stakkevollan, 9037 Tromsø, Norway

**Keywords:** Malnutrition, Frailty, Sarcopenia, Obesity, Dehydration

## Abstract

**Objectives:**

To investigate the overlap between the nutrition disorders (malnutrition, low-intake dehydration, obesity) and nutrition related conditions (frailty, sarcopenia, sarcopenic obesity), and the significance of each of these and their combinations for survival among older patients admitted to geriatric care.

**Methods:**

This exploratory study was based on a cross-sectional study with 100 patients (≥65 years) admitted to two geriatric departments. Data was retrieved from the Electronic Patient Record. Malnutrition was defined according to the Global Leadership Initiative on Malnutrition (GLIM) criteria with no prior screening. A low-intake dehydration equation, using proxy urea, was applied. Obesity was diagnosed at BMI > 29.9 kg/m^2^. Frailty was assessed by Clinical Frailty Scale, whereas sarcopenia was diagnosed according to the European Working Group of Sarcopenia in Older People (EWGSOP2). Sarcopenic obesity was defined as the combination of sarcopenia and obesity. Mortality was recorded up to one year after discharge.

**Main findings:**

The nutrition disorders and nutrition related conditions displayed considerable overlaps, and the prevalences were; frailty 67%, low-intake dehydration 62%, malnutrition 58%, sarcopenia 30%, obesity 13%, and sarcopenic obesity 0%. Higher numbers of nutrition disorders and nutrition related conditions combined, and malnutrition (according to GLIM) alone, were related to decreased one-year survival as show in Kaplan Meier plots.

**Conclusion:**

The prevalence and overlap of the nutrition disorders; malnutrition, and low-intake dehydration and the nutrition related conditions; frailty and sarcopenia were high in patients acutely admitted to geriatric departments. Increasing number of nutrition disorders and nutrition related conditions combined, and malnutrition alone were associated with decreased survival.

## Graphical abstract

**Figure:**
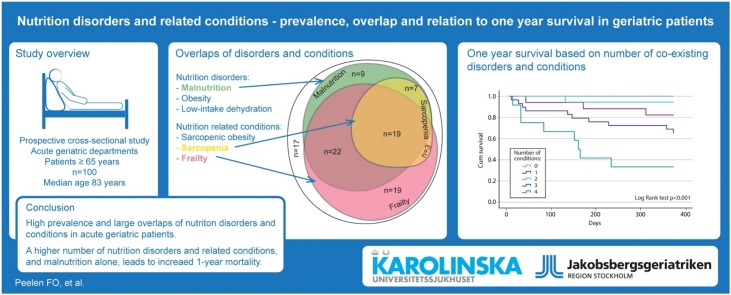

## 1 Introduction

Older adults have an increased risk of nutrition disorders such as malnutrition, obesity and dehydration, and of nutrition related conditions such as frailty and sarcopenia [1]. These disorders and conditions are often associated with poor outcomes, e.g. increased rates of infections and duration of convalescence as well as shorter survival [2]. In addition, dehydration is also related to serious health consequences [1]. On the other hand, obesity with its well-known negative health consequences is an increasing problem also in older adults [1], although obese older patients seem to have an increased survival [3].

In recent years, there has been important initiatives to reach international consensus for the diagnoses of malnutrition [5], sarcopenia [6], and dehydration [1,7], and for the understanding of frailty [8]. Frequently used definitions of frailty are the criteria from Fried *et al*. [9] which include physical health and the Clinical Frailty Scale (CFS) which account for both the physical and cognitive health. Several criteria for sarcopenia also exist, however the European Working Group on Sarcopenia in Older People (EWGSOP) [6] criteria are frequently used.

Malnutrition is regarded as one important contributing factor in the complex etiology of sarcopenia and frailty [1]. Accordingly, overlaps between these three nutrition disorders/conditions are expected due to their partly common etiology with inflammation as an important driver. For older hospitalized patients at risk of malnutrition, individual nutrition support has beneficial effects on long-term survival, functional outcomes and quality of life measures [12,13]. Furthermore, nutritional support seemed effective in patients with age-related frailty [12], but less effective in patients who were sarcopenic [14], and in those patients with the highest degree of inflammation [15].

It is hence crucial to identify patients with nutrition disorders and nutrition related conditions and their overlap, in order to understand their common etiology and target effective treatments [16]. Thus, the first aim of the current study was to investigate the prevalence and overlap between the nutrition disorders and nutrition related conditions, whereas the second aim was to evaluate the significance these disorders and conditions for survival among older patients admitted to geriatric care.

## 2 Methods

### 2.1 Study design and ethics

This is a secondary analysis based on data from a previously described cross-sectional study [4] with 100 prospectively recruited patients from two acute geriatric wards at Karolinska University Hospital and the Jakobsbergs Hospital, Sweden.

The objectives of the original study were to describe the prevalence of malnutrition based on the GLIM criteria, the impact of using various screening tools for the prevalence of malnutrition, and to evaluate the feasibility of the GLIM criteria in geriatric inpatients. The study was approved by the Swedish Ethical Review Authority (Dnr 2022-01822-01), and followed the Strengthening the Reporting of Observational Studies in Epidemiology (STROBE) guidelines [17]. Additional details on study design can be found elsewhere [4]. For the present study approval of collection of complementary data from the Electronic Patient Record (EPR) was achieved from the Swedish Ethical Review Authority (Dnr 2024-01773-02).

### 2.2 Participants and recruitment

To be eligible, participants were required to be ≥65 years, a life expectancy of >3 months, and be able to consent (for example speaking Swedish or not having severe cognitive impairment). All participants received written and verbal study information, and participation was voluntary and based on written consent.

### 2.3 Data collection

Variables including baseline characteristics were extracted from the EPR after inclusion. In addition, physical therapists or registered nurses at the wards measured hand grip strength, calf circumference (CC) and Mid Arm Circumference (MAC) (see below) and documented the results in the EPR.

Information regarding sex, age, BMI, CRP, CC, MAC, hand grip strength and malnutrition according to GLIM, was already available [4]. Complementary information regarding: living situation at admission, plasma-albumin, plasma-hemoglobin, plasma-sodium, plasma-potassium, plasma-glucose, plasma-creatinine, frailty, risk for pressure ulcers, risk for falls, actual falls during the hospital stay, hydration status, length of stay (LOS) and mortality was collected from the EPR by the researchers after extended ethical approval.

### 2.4 Definitions of nutrition disorders

#### 2.4.1 Malnutrition

The GLIM criteria were used for the diagnosis of malnutrition, with no prior screening. The diagnosis of malnutrition by GLIM was confirmed by fulfilling any combination of at least one phenotypic criterion; i.e., weight loss, low body mass index (BMI) or low muscle mass, and at least one etiologic criterion; i.e., reduced food intake/assimilation or inflammatory disease burden. All GLIM criteria were evaluated, and cut-off values for weight loss and BMI were used as defined in GLIM [5,18]. The GLIM criteria were feasible for this population, for details see reference [4].

Briefly, low muscle mass was defined as calf circumference <33 cm for men and <32 cm for women [19]. If BMI was 25–30 kg/m^2^ the measured value was reduced by 3 cm, and if BMI was above 30 kg/m^2^ the measured value was reduced by 7 cm [19]. For patients with oedema in lower extremities, mid-upper arm circumference was used with a cut-off of <21 cm for both sexes [20].

Food intake was based on a standardized food record for the first three days of admission. Energy requirements were estimated as 25 kcal/kg if bed-ridden and 30 kcal/kg if able to move around and adjusted if BMI was >25 kg/m^2^ [4]. Information from EPR about dysphagia, nausea/vomiting, diarrhea and constipation was used to assess food assimilation.

Disease burden/inflammation was based on clinical diagnosis of acute and chronic inflammatory diseases extracted from EPR, while plasma C-reactive protein (CRP) > 10 mg/L [15] was used as a supportive measure in cases of uncertainty.

#### 2.4.2 Obesity

Based on the WHO definition, obesity was defined as BMI above 29.9 kg/m^2^ [21].

#### 2.4.3 Low-intake dehydration

Low-intake dehydration is not described as either a nutrition disorder or nutrition related condition in the ESPEN terminology paper, but due to its cause of low-intake we decided to classify it as a nutrition disorder. The equation: 1.86 x (Sodium + Potassium) + 1.15 x glucose + urea + 14 (all measured in mmol/L) with an action threshold of >295 mmol/L was used for the definition of low-intake dehydration as recommended by [1]. Since no urea measure was available a proxy urea (the highest normal range for the age group) 7.9 mmol/L for women and 8.2 mmol/L for men was used. These proxy values are similar to urea concentrations in old patients admitted to medical care/emergency departments as noticed in a study with similar kinds of patients [22].

### 2.5 Definition of nutrition related conditions

#### 2.5.1 Frailty

No information was available from the EPR for the assessment of physical frailty. Instead, the CFS was used to estimate degree of frailty. CFS is a nine-degree graded scale, based on clinical judgment, from grade 1 (“very fit”) to grade 9 (“terminally ill”) assessed as the *“baseline health state”* [10] defined as the functional level two weeks before the current examination; e.g. hospital admission [10,23]. CFS grade 5 or more is generally considered a cut off for frailty [23]. The CFS grading was performed at admission at the Karolinska University Hospital by the physicians, whereas at the Jakobsberg site CFS grading was decided by the team conference including the physician, nurse and the rehabilitation staff, within the first 2–3 days after hospital admission.

If CFS was missing in the EPR, the researchers FOP and ME registered CFS grade 5 if the patients were dependent on home care service, which is in accordance with the definition of living with mild frailty (CFS 5): “*more evident slowing and need help in high order in IADL (Instrumental Activities of Daily Life). Typically, mild frailty progressively impairs shopping and walking outside alone, meal preparation and housework*” [10].

#### 2.5.2 Sarcopenia

For the definition of sarcopenia, the second version of the European Working Group on Sarcopenia in Older People (EWGSOP2) [6] definition was used. Strength was assessed by hand grip and muscle mass was evaluated using calf circumference as a proxy [24]. Cut off values of <33 cm for men, and <32 cm for women was based on recommendations from the GLIM Consortium [24]. If overweight or obesity was present, the measured value was reduced by 3 cm if BMI was 25–30 and by 7 cm if BMI was above 30 kg/m^2^. If a patient had oedema (based on clinical observation) in lower extremities, MAC was measured using a tape at the midpoint of the upper arm instead. MAC less than 21 cm was defined as low muscle mass for both sexes [20].

#### 2.5.3 Sarcopenic obesity

ESPEN and the EASO (European Association for the Study of Obesity) have published a consensus paper on how to assess sarcopenic obesity [25]. However, since several of the recommended assessments were not available from EPR sarcopenic obesity was defined as the co-occurrence of sarcopenia (as defined above) and obesity (as defined above).

### 2.6 Other variables

In order to have more detailed characteristics of the study population the following data were collected:

#### 2.6.1 Risk of pressure ulcers

The risk of pressure ulcer was evaluated by the modified Norton scale [26], compiled of seven questions (1–4 points each, in total range of 7−28 points). The nurses’ registration in the EPR during the first day of the stay was used; where 20 points or less indicated increased risk of pressure ulcers.

#### 2.6.2 Risk of falls during the hospital stay

The risk of falls was evaluated by the Downton fall risk index (0−11 points) [27], based on questions regarding previous falls (0–1 points), medications (0–5 points), sensory impairments (0–3 points), cognitive impairments (0–1 points), walking ability (0–1 points). The nurses’ registration in the EPR during the first day of the stay was used; where 3 points or more indicated increased risk for falls. Actual falls and related injuries during the hospital stay were extracted from the EPR.

### 2.7 Outcome

The primary outcome of the present study was survival. In order to assess survival, mortality up to 1 year after discharge was retrieved from the EPR which is linked to the National Cause of Death Register of Sweden.

### 2.8 Sample size and statistics

For the primary analyses of prevalence of malnutrition (published in Ref. [4]), sample size was estimated to 98.

Data was handled and analyzed using SPSS and Microsoft Excel. Continuous variables are presented as median and range since none were normally distributed. Venn diagrams which illustrate both prevalence and overlap of conditions, were created in Microsoft Excel and Adobe Illustrator. Kaplan-Meier plots were used to estimate overall survival of patients with and without the nutrition disorders and nutrition related conditions; as well the co-existence of multiple disorders/conditions. The differences in survival were analyzed with the log-rank test. A *p*-value of <0.05 was considered statistically significant.

## 3 Results

A total of 243 patients were admitted during the project period. Based on the given inclusion and exclusion criteria, 118 patients were asked to participate, and 100 consented to participation. Flow of inclusion is previously published [4]. Baseline characteristics for the total population (*n* = 100) are presented in Table 1.

**Table 1:** Characteristics of the population.

	Total population (*n* = 100)
Sex, *n* (%)	
Women	65 (65)
Age (years), median (range)	83 (65−96)
Length of stay (days), median (range)	7 (3−35)
Plasma albumin (g/L) (*n* = 63), median (range)	30 (16−37)
(Reference values: 40−68 years: 36−45 g/L; ≥70 years: 34−45 g/L)	
Plasma C-reactive protein (CRP) (mg/L) (*n* = 98), median (range)	25 (1−316)
Above cut-off (>10 mg/L), n (%)	68 (68)
Hemoglobin (g/dL) (*n* = 96), median (range)	114 (68−180)
(Reference values: 117−153 g/dL (women); 134−170 g/dL (men))	
Plasma Creatinine (μmol/L) (*n* = 97), median (range)	78 (19−619)
(Cut-off: <90 μmol/L (women); <100 μmol/L (men))	
Living situation at admission	
Home-living without services, *n* (%)	54 (54)
Home-living with home care service, *n* (%)	44 (44)
Short residential care facility, *n* (%)	1 (1)
Residential care facility, *n* (%)	1 (1)
Modified Norton scale (7−28 p) (*n* = 99), median (range)	23 (14−27)
Risk of pressure ulcers (≤20 p), *n* (%)	22 (22)
Downton fall risk index (0−11 p) (*n* = 97) median (range)	4 (0−8)
Risk of falls (≥3 p), *n* (%)	75 (77)
Falls during hospitalization	
Fall without injury, *n* (%)	6 (6)
Fall with injury, *n* (%)	1 (1)
Energy intake	
Below 75% of estimated needs, *n* (%)	43 (43)
Below 50% of estimated needs, *n* (%)	4 (4)
Provision of enteral nutrition, *n* (%)	1 (1)

### 3.1 Frequency of nutrition disorders and nutrition related conditions

The frequency of the six included nutrition disorders and nutrition related conditions is listed in Table 2. The most frequent condition was frailty, followed by malnutrition and low-intake dehydration. Details about the laboratory values used to calculate the prevalence of low-intake dehydration can be found in Appendix 1, Table 1. Further details on each of the GLIM criteria can be found in Enge et al. [4].

**Table 2:** Frequency of nutrition disorders and nutrition related conditions.

Nutrition disorders and nutrition related conditions	Frequency of disorder/condition
**Malnutrition (GLIM) (*n* = 100), *n* (%)**	**58 (58)**
BMI, median, (range)	24 (14−40)
Overweight (25−29,9), *n* (%)	24 (24)
**Obesity (BMI > 29.9) (*n* = 100), *n* (%)**	**13 (13)**
Hydration (*n* = 84), median (range) mmol/L^c^	297 (266−322)
**Low-intake dehydration (osmolarity >295) (*n* = 84), *n* (%)**	**52 (62)**
Clinical Frailty Scale (CFS) (*n* = 81) median (range)	5 (2−8)
Frailty according to CFS (*n* = 81), n (%)	57 (70)
Frailty according to imputed CFS (*n* = 19), *n* (%)^a^	10 (53)
**Total frailty**^b^**(*n* = 100), *n* (%)**	**67 (67)**
**Sarcopenia (*n* = 96), *n* (%)**	**29 (30)**
**Sarcopenic obesity (*n* = 96), *n* (%)**	**0 (0)**

Main disorder/conditions in bold.

a If CFS was missing in the EPR, CFS grade 5 was registered if the patients were dependent on home care service, which is in accordance with the definition of living with mild frailty (CFS 5): “*more evident slowing and need help in high order in IADL (Instrumental Activities of Daily Life). Typically, mild frailty progressively impairs shopping and walking outside alone, meal preparation and housework*” (5).

b Total frailty = Frailty according to CFS + Frailty according to imputed CFS.

c The equation: 1.86 × (Na + Ka) + 1.15 × glucose + proxy urea 7.9/8.2 + 14 (all measured in mmol/L) with an action threshold of >295 mmol/L was used for Modified dehydration score.

### 3.2 Overlap between nutrition disorders and nutrition related conditions

**Fig. 1:**
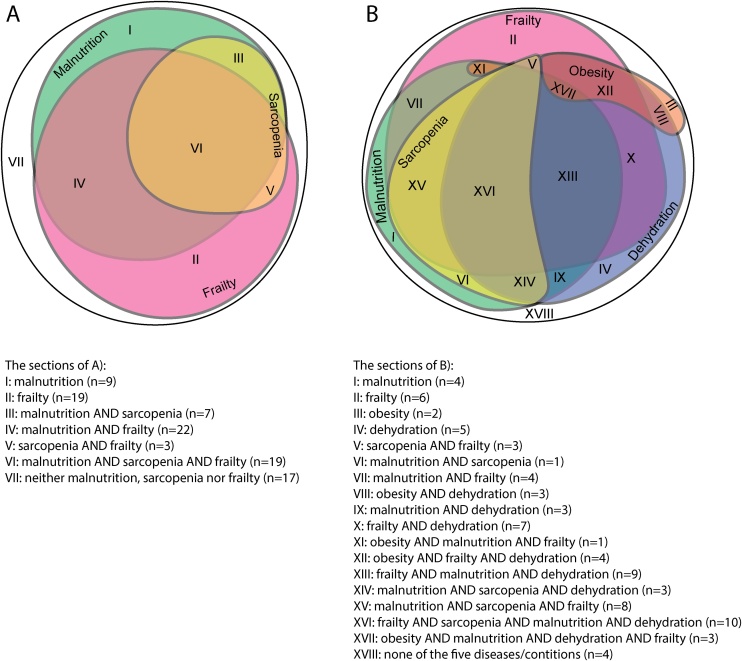
Overlap and prevalence of nutrition disorders and nutrition related conditions. Each colored area represents one disorder or condition with the size reflecting the prevalence, whereas the overlap illustrates co-existing disorders or conditions. Each section is numbered by roman numerals and listed below the diagram. A) Venn diagram for the prevalence and overlap of nutrition disorder; malnutrition, and nutrition related conditions; frailty and sarcopenia (*n* = 96). B) Venn diagram for the prevalence and overlap between nutrition disorders; malnutrition, obesity, low-intake dehydration (“Dehydration” in figure) and nutrition related conditions; frailty, and sarcopenia (*n* = 80).

The Venn diagram in Fig. 1A shows the overlap between malnutrition, sarcopenia and frailty for the patients with complete dataset for all three disorders/conditions (*n* = 96). All three disorders/conditions were present in 19 patients (20 %), while the most frequent combination of disorders/conditions was frailty and malnutrition (23 %). There was an almost complete overlap for sarcopenia with malnutrition (90%) with only 3 patients having sarcopenia, but not being malnourished. However, 32 patients were categorized as malnourished without having concurrent sarcopenia. For frailty there was less overlap with malnutrition as compared to sarcopenia. Of the 63 patients categorized as frail, 19 did not overlap with either malnutrition or sarcopenia.

Seventeen patients had neither malnutrition, sarcopenia nor frailty. Their median was 78 years, they had normal to high BMI (23−34 kg/m^2^), a median 4 days hospitals stay (range 3–10 days), and on average 4 comorbidities. Sixteen lived at home without home care services and were discharged back to home without services. Only one of the 17 patients with neither frailty, malnutrition nor sarcopenia died within one year.

Fig. 1B shows the overlap between the nutrition disorders; malnutrition, obesity, and low-intake dehydration and the nutrition related conditions; sarcopenia, and frailty, for the 80 patients with complete dataset for all five disorders/conditions. Details of specific numbers can be found in Appendix 1, Table 2. None of the patients had all five disorders/conditions concurrently. The majority of patients (*n* = 59, 74%) had two or more of the five disorders/conditions. More specifically, 18 patients (23%) had two, 28 (35%) patients had three, and 13 (16%) patients had four concurrent disorders/conditions.

### 3.3 Nutrition disorders and nutrition related conditions and survival

**Fig. 2:**
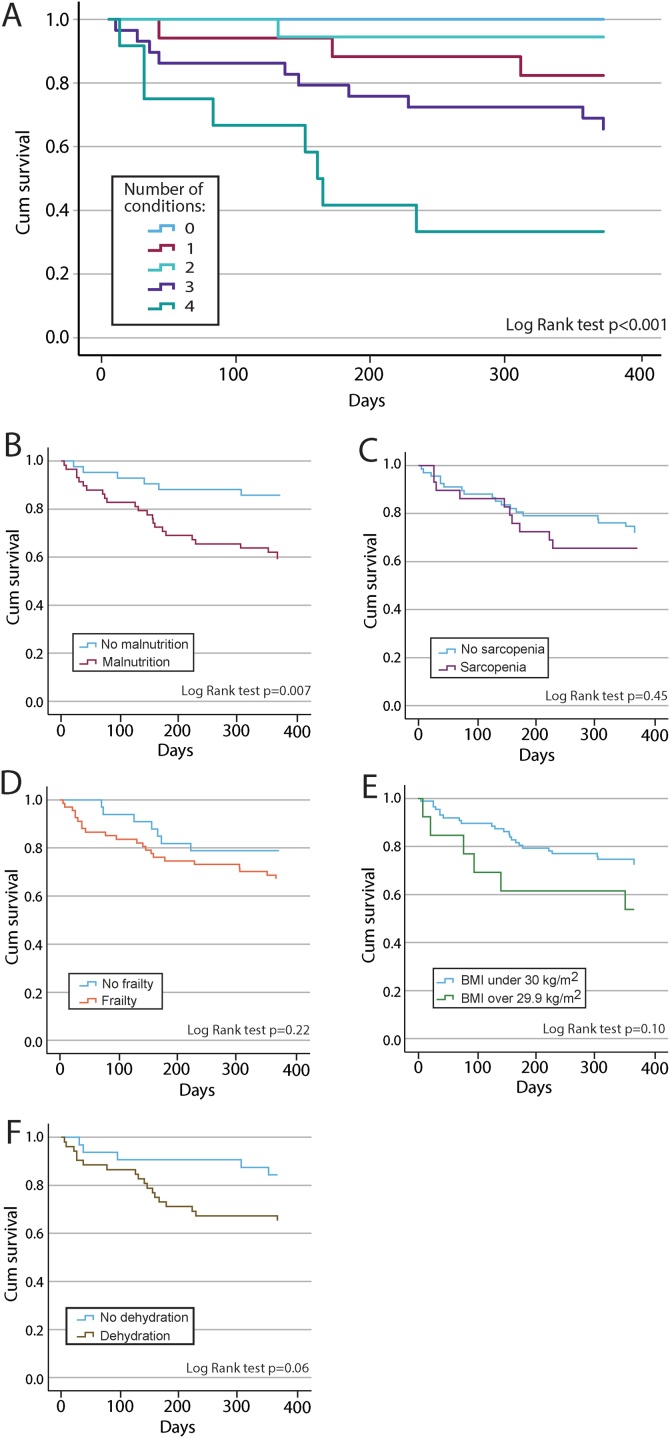
Kaplan Meier for 1 year survival based on the number of nutrition disorders and conditions, and for each nutrition disorder and nutrition related condition separately. A) The number of nutrition disorders and conditions, B) Malnutrition, C) Sarcopenia, D) Frailty, E) Obesity, F) Low-intake dehydration (“Dehydration” in figure).

Of 80 patients with complete datasets for all disorders/conditions, 22 (28%) patients died within one year, i.e.; two (3%) during the hospital stay, five (6%) within one month, five (6%) within one to three months and 10 (13%) within three months to one year (Fig. 2A). There was a higher risk of death with an increasing combined number of disorders/conditions (Log rank test *p* < 0.001). Of the diseased patients, 100 % fulfilled the inflammatory disease burden in GLIM criterion and 72 % had a low muscle mass.

The thirteen patients with four nutrition disorders/conditions were 79 years (median), eleven women and two men. All thirteen fulfilled the inflammation disease burden criterion in GLIM with a CRP median of 37 mg/L. All thirteen were malnourished, frail and dehydrated, ten had sarcopenia and three had obesity.

Next, survival was investigated for each of the disorders/conditions malnutrition, sarcopenia, frailty, obesity and low-intake dehydration separately (Fig. 2B–F). The highest one-year mortality was observed in the patients with malnutrition (*P* = 0.007) (Fig. 2B). Furthermore, there was a trend towards higher mortality among the patients with low-intake dehydration (*P* = 0.06) (Fig. 2F). In this sample, none of the nutrition related conditions had a statistically significant association with one-year survival.

## 4 Discussion

### 4.1 Main findings

This study found that there is a high degree of overlap of nutrition disorders and nutrition related conditions in patients admitted to geriatric care. Furthermore, the majority of patients in the acute geriatric wards (74%, 59/80) had two or more disorders and conditions concurrently. In addition, we found an association between shorter survival and malnutrition, as well as with increasing numbers of overlapping disorders/conditions. None of the other nutrition related disorders alone had a statistically significant impact on survival, possibly given that the sample of patients was quite small.

### 4.2 Overlap of nutrition disorders, nutrition related conditions and survival

According to our knowledge no former studies have simultaneously assessed the risk of mortality for these five overlapping nutrition disorders and related conditions among older patients. However, in the prospective UK Biobank study among middle-aged as well as older people, the risk of mortality increased five-fold for those with frailty (by the Fried criteria) in combination with two or more of the conditions or disorders (sarcopenia, malnutrition, cachexia). Frailty had the highest prevalence (45%) and was present in 92 % of people with malnutrition, and in everyone with sarcopenia or cachexia. In our population, we did not assess cachexia specifically, however cachexia is a subcategory of malnutrition (i.e. disease-related malnutrition with inflammation) [28], and thus should be considered in our malnourished patients.

The overlap between malnutrition, frailty, and sarcopenia can be partly attributed to their shared etiology. However, we and other studies [11,29] find that this overlap is not uniform across all patients, raising a paradox regarding the assessment of these disorders and conditions in busy hospital settings where prioritization is crucial due to limited resources. Interestingly, we found that malnutrition was correlated with mortality, indicating that malnutrition screening and assessment needs to be prioritized in geriatric patients. Secondly, evaluation of malnutrition should be prioritized since there are well established treatment options to reduce the consequences of malnutrition, and nutrition support is also effective in patients with age-related frailty [12]. Moreover, nutritional care is considered a human right [30]. The GLIM criteria is a comprehensive diagnostic technique as it encompasses a broad range of criteria, including weight loss, BMI, muscle mass, food intake or assimilation as well as inflammation disease burden [5]. GLIM includes elements from both sarcopenia and frailty and may facilitate a more thorough evaluation of these interrelated disorders/conditions. Nonetheless, further research is essential to explore the distinct impact of the high prevalence of malnutrition, frailty, and sarcopenia on other clinical endpoints in this patient population such as health-related quality of life, functional capabilities, readmissions, and morbidity, also including the significance of dehydration. Understanding these differences will guide healthcare providers in effectively prioritizing care for acutely admitted geriatric patients, ensuring that interventions are targeted where they can have the most significant impact.

### 4.3 Survival, nutrition disorders and nutrition related conditions

Malnutrition was the only of the five nutrition conditions and nutrition related conditions with a significantly shorter survival in this study of one hundred patients. This occurrence of shorter survival has been documented by others as well. Sobestiansky *et al*. [31] found that malnutrition according to GLIM was related to increased 1-y mortality among older patients. Hirose *et al*. [32] showed that malnutrition according to GLIM among old patients with heart failure, was associated with higher mortality rate independent of other prognostic factors. A retrospective cohort study in 9372 hospitalized old patients in Japan showed that malnutrition was an independent risk factor for shorter survival after adjusting for age and sex, and that all GLIM criteria, besides BMI, were independently associated with prognosis [33]. Hence there might be specific GLIM criteria impeding survival more than others. The burden of disease-related inflammation or low muscle mass (measured by CC or MAC) included in the GLIM criteria might have been main contributors to the shortened survival observed in our study since 100% of the deceased patients fulfilled the inflammatory disease burden criterion in GLIM and 72% had low muscle mass.

Even though it was not statistically significant (*p* = 0.06), our findings indicate that low-intake dehydration might shorten the survival. This is supported by a systematic review of studies of dehydration among participants 60 years or older, recruited from hospital settings, medical long-term care centers and the community dwelling population [34]. In the studies including hospitalized patients, the 30-day mortality rates were over twice as high in the dehydrated patients [34]. Dehydration was an important indicator contributing to malnutrition in old people receiving home care [35], in accordance with our high degree of overlap between the two disorders. In spite of the high prevalence (62%) of low-intake dehydration our study probably did not have statistical power to detect this potential decrease in survival.

Previous studies have documented an obesity paradox among older patients; i.e. the patients with obesity seemed to have an increased survival [3]. We were not able to confirm this in our population. However, only 13 patients were obese. Furthermore, none of the patients had the nutrition related condition sarcopenic obesity. Six of the 13 obese patients had low hand-grip strength, and thus we speculate that the anthropometric cut offs for muscle mass underestimate sarcopenia for this subgroup. Also, our way of assessing sarcopenic obesity, which was not according to the consensus from ESPEN and EASO [25] might have been less accurate.

In this small study, the nutrition related condition frailty was not significantly associated with decreased survival. This is in contrast with a meta-analysis assessing the CFS as a predictor of short-term mortality in patients admitted to emergency departments [36]. These authors found an increased mortality both at the hospital and at the 1-month follow-up among those with a CFS at or above 5. Our study is relatively small, with a very high prevalence of frailty. Thus, our study might not be powered to detect a difference in mortality between the frail and the few non-frail. In addition, we did not see a decreased survival among patients with sarcopenia; i.e. the other nutrition related condition. This may again be due to the limited number of patients with this condition.

### 4.4 Strengths and weaknesses

A strength of our study is the concurrent assessment of several prevalent age-related disorders and conditions: i.e., malnutrition, obesity and low-intake dehydration, sarcopenia, frailty and sarcopenic obesity. Some study limitations are important to mention. Firstly, since this study is a secondary analysis, it might not be powered to fully address its aims, which may affect the robustness of the findings. Secondly, we estimated patients' hydration status using a modified dehydration equation based on a proxy of urea as no biobank was available. The effect of this proxy urea on the prevalence of dehydration is uncertain, however it is unlikely to have a large impact since urea makes a minor contribution to the entire equation. Another weakness is that frailty evaluation by CFS score was lacking for 19 of the patients, and consensus-based decisions about frailty were made by the researchers based on the available data. Since this alternative frailty decision was based on alternative data for dependency and function, it is unlikely to have significantly influenced the prevalence of frailty. Also, CFS was used rather than a measure of physical frailty. CC or MAC were used as a proxy for muscle mass due to the unavailability of direct muscle mass measurements. Finally, the data were drawn from the EPR and there are both strengths and limitations of such real-world data reflecting clinical practice.

## 5 Conclusion

The prevalence of the nutrition disorders; malnutrition, and low-intake dehydration and the nutrition related conditions; frailty and sarcopenia were high in this population of patients acutely admitted to geriatric departments, with considerable overlap between all disorders and conditions. An increasing number of nutrition disorders and nutrition related conditions combined, and malnutrition alone were associated with decreased survival. With increasing awareness of all nutrition disorders and related conditions, our findings emphasize that continuing screening and assessment of malnutrition should be prioritized in order to provide adequate and individualized treatment.

## Ethical standards

The study was approved by the Swedish Ethical Review Authority (Dnr 2022-01822-01 and 2024-01773-02) and was conducted according to the guidelines of the Helsinki Declaration. Informed consent was obtained from all individual participants included in the study.

## Informed consent statement

All participants have received verbal and written information and written informed consent was obtained before enrollment.

## Declaration of Generative AI and AI-assisted technologies in the writing process

During the preparation of this work no AI were used.

## Funding

This research did not receive any specific grant from funding agencies in the public, commercial, or not-for-profit sectors. Karolinska University Hospital, Sweden funded the ethical application.

## CRediT authorship contribution statement

**Frida Ostonen Peelen:** Conceptualization, Methodology, Formal analysis, Investigation, Data curation, Project administration, Writing - original draft. **Maria Enge:** Conceptualization, Methodology, Formal analysis, Investigation, Data curation, Project administration, Writing - original draft. **Rikke Lundsgaard Nielsen:** Conceptualization, Methodology, Formal analysis, Data curation, Project administration, Writing - original draft. **Anne Marie Beck:** Conceptualization, Methodology, Formal analysis, Data curation, Project administration, Writing - original draft, Supervision. **Ann Ödlund Olin:** Conceptualization, Methodology, Project administration, Writing - original draft, Supervision. **Tommy Cederholm:** Conceptualization, Project administration, Resources, Writing - review & editing. **Anne-Marie Boström:** Project administration, Resources, Writing - review & editing. **Ingvild Paur:** Conceptualization, Methodology, Formal analysis, Data curation, Project administration, Visualization, Writing - original draft.

## Declaration of competing interest

All authors declare no conflict of interest.
