# Supplementary material for: Nutrition disorders and related conditions—Prevalence, overlap and relation to one year survival in geriatric patients
Source: J Nutr Health Aging. 2025 Sep 20;29(11):100682. doi: 10.1016/j.jnha.2025.100682 (PMC12489919; doi:10.1016/j.jnha.2025.100682)
Supplement: Supplementary file 1 [file mmc1.pdf]

Supplementary tables.

Peelen *et al*. Nutrition disorders and related conditions – prevalence, overlap and relation to one year survival in geriatric patients

Supplementary table 1. Laboratory values (for the 80 patients with complete dataset for all five nutrition disorders and nutrition related conditions) used for calculation of low intake dehydration according to the equation: 1.86 x (Sodium + Potassium) + 1.15 x glucose + urea + 14 (all measured in mmol/L). Proxy urea (the highest normal range for the age group) 7.9 mmol/L for women and 8.2 mmol/L for men was used. Of these 80 patients, 54 (67.5%) female and 26 (32.5%) were male patients.

**Laboratory value**	**Median, (range)**
Plasma sodium (mmol/L)<br>(Reference values 137-145 mmol/L)	139 (124-152)
Plasma potassium (mmol/L)<br>(Reference values 3.5-4.6 mmol/L)	4 (2.9-4.9)
Plasma glucose (mmol/L)<br>(Reference values 4.1-6.0 mmol/L)	7 (4.3-14)

Supplementary table 2. Overlap of nutrition conditions and nutrition related diseases (The combinations of two or more that do not exist, are not reported).

**Nutrition disorder/ Nutrition related condition**	**Number (%) of 80 with all data available**
No diagnose	4 (5)
Malnutrition	4 (5)
Sarcopenia	0 (0)
Frailty	6 (7.5)
Obesity	2 (2.5)
Dehydration (D)	5 (6.3)
Malnutrition + Sarcopenia	1 (1.3)
Malnutrition + Frailty	4 (5)
Malnutrition + Dehydration	3 (3.8)
Frailty + Dehydration	7 (8.8)
Obesity + Dehydration	3 (3.8)
Malnutrition + Sarcopenia + Frailty	9 (11.3)
Malnutrition + Frailty + Dehydration	9 (11.3)
Malnutrition + Frailty + Obesity	1 (1.3)
Malnutrition + Sarcopenia + Dehydration	1 (1.3)
Sarcopenia + Frailty + Dehydration	4 (5)
Frailty + Obesity + Dehydration	4 (5)
Malnutrition + Frailty + Obesity + Dehydration	3 (3.8)
Malnutrition + Frailty + Sarcopenia + Dehydration	10 (12.5)
